# Multimorbidity and determinants for initiating outpatient trajectories: A population-based study

**DOI:** 10.1186/s12889-023-15453-w

**Published:** 2023-04-21

**Authors:** Cathrine Bell, Anders Prior, Charlotte Weiling Appel, Anne Frølich, Asger Roer Pedersen, Peter Vedsted

**Affiliations:** 1 Diagnostic Centre - University Research Clinic for Innovative Patient Pathways, Silkeborg Regional Hospital, Central Denmark Region, Silkeborg, Danmark; 2 Research Unit for General Practice, Aarhus, Denmark; 3 Innovation and Research Centre for Multimorbidity, Slagelse Hospital, Region Zealand, Denmark; 4 Centre for General Practice, Faculty of Health and Medical Sciences, University of Copenhagen, Copenhagen, Denmark

**Keywords:** Denmark, Outpatient, Hospital, Multimorbidity, Healthcare utilization, Trajectory

## Abstract

**Introduction:**

Individuals with multimorbidity often receive high numbers of hospital outpatient services in concurrent trajectories. Nevertheless, little is known about factors associated with initiating new hospital outpatient trajectories; identified as the continued use of outpatient contacts for the same medical condition.

**Purpose:**

To investigate whether the number of chronic conditions and sociodemographic characteristics in adults with multimorbidity is associated with entering a hospital outpatient trajectory in this population.

**Methods:**

This population-based register study included all adults in Denmark with multimorbidity on January 1, 2018. The exposures were number of chronic conditions and sociodemographic characteristics, and the outcome was the rate of starting a new outpatient trajectory during 2018. Analyses were stratified by the number of existing outpatient trajectories. We used Poisson regression analysis, and results were expressed as incidence rates and incidence rate ratios with 95% confidence intervals. We followed the individuals during the entire year of 2018, accounting for person-time by hospitalization, emigration, and death.

**Results:**

Incidence rates for new outpatient trajectories were highest for individuals with low household income and ≥3 existing trajectories and for individuals with ≥3 chronic conditions and in no already established outpatient trajectory. A high number of chronic conditions and male gender were found to be determinants for initiating a new outpatient trajectory, regardless of the number of existing trajectories. Low educational level was a determinant when combined with 1, 2, and ≥3 existing trajectories, and increasing age, western ethnicity, and unemployment when combined with 0, 1, and 2 existing trajectories.

**Conclusion:**

A high number of chronic conditions, male gender, high age, low educational level and unemployment were determinants for initiation of an outpatient trajectory. The rate was modified by the existing number of outpatient trajectories. The results may help identify those with multimorbidity at greatest risk of having a new hospital outpatient trajectory initiated.

**Supplementary Information:**

The online version contains supplementary material available at 10.1186/s12889-023-15453-w.

## Plain Language Summary

Many people with multiple chronic diseases (multimorbidity) need to see a doctor regularly. They must often go to several different hospital units for check-ups and medical examinations, and these consultations are rarely coordinated to provide integrated care. This means that these patients are often treated in parallel trajectories in several specialized outpatient clinics, and many receive fragmented care. This study investigates determinants for having a new ‘care trajectory’ initiated. It documents that men have a higher rate than women of initiating a new trajectory and that the rate increases with higher age, more chronic conditions, and low income. These results can be used to identify those at greatest risk of entering an additional trajectory, who may require special attention. Such information will enable clinicians to offer better, planned outpatient services and perhaps also more coherent care.

## Introduction

An increasing number of people are living with multimorbidity (coexistence of two or more chronic conditions) [1]. This development is due to aging populations and improved treatment of chronic conditions [2–6]. Most individuals with multimorbidity are managed in general practice, but some require additional care in the form of outpatient specialized services delivered by hospitals [7–11]. However, research shows that it is difficult for patients and healthcare professionals to navigate healthcare plans and numerous appointments [12, 13]. Multiple chronic conditions can lead to concurrent use of outpatient clinics [7, 8], which may lead to high healthcare utilization, less integrated health care, low medical quality, and low patient satisfaction [9, 11, 14, 15]. Substantial utilisation of outpatient attendances can add to the healthcare workload and the impact on well-being experienced by individuals with multimorbidity, known as treatment burden. This can negatively affect quality-of-life and adherence to treatments [16, 17].

Multimorbidity is associated with socioeconomic deprivation [15, 18], which has been linked with lower probability of specialist visits [14, 19, 20]. While former studies have described disease patterns, demographics, and social disparities according to outpatient contacts and hospitalizations [14, 15, 19, 21, 22], no evidence exists on factors for entering hospital outpatient trajectories studied as the continued use of outpatient contacts. Multimorbidity trajectories have often been studied as change or accumulation in the number of distinguishable conditions [23] but can also be regarded as the patient’s way through the healthcare system considered as a series of events [24, 25]. The benefits of receiving interprofessional health care have been widely recognized, particularly in complex patient trajectories [1]. Identifying determinants for initiation of trajectories in specialized outpatient clinics may promote proactive targeting of services and facilitate collaboration between specialists.

This study aimed to investigate the number of chronic conditions and sociodemographic characteristics as determinants of initiating a hospital outpatient trajectory in adults with multimorbidity.

## Materials and Methods

### Setting

A population of 5.8 million people are residing in Denmark [26]. The healthcare system is mostly funded through taxation, and residents have free access to general practitioners and to public hospitals after referral. Nearly all Danish residents (99%) are registered with a general practitioner (GP). The GP is the patient’s first point of contact to the healthcare system and serves as gatekeeper to specialized care through a referral system. For each healthcare encounter, data is routinely collected and stored in national registers. All Danish residents are provided with a unique 10-digit civil registration number at birth or immigration. This number enables individual-level linkage between registers. Information about residence status, vital status, and migration with long-term follow-up can be obtained from the Danish Civil Registration System (CRS) through the civil registration number [27–29].

### Design

This population-based cohort study was based on data from adult Danish residents identified with multimorbidity in Danish registers at the index date, January 1, 2018. Study participants were divided into four categories of existing hospital outpatient trajectories (0, 1, 2, or ≥3) at the index date, then followed prospectively from January 1, 2018, to December 31, 2019.

### Study participants

The CRS was used to identify all legal residents aged 18 years or more residing in Denmark on January 1, 2018. We linked the civil registration number to diagnoses recorded in the Danish National Patient Register (NPR), the Danish Psychiatric Central Register (DPCR), or the Danish National Prescription Registry (DNPR). The NPR contains information on all inpatient and outpatient visits to somatic hospitals, including diagnoses [30]. The DPCR covers similar information for psychiatric hospitals [31]. Medical conditions recorded according to the International Classification of Diseases 10th version (ICD-10) were extracted from the NPR (inpatient visits from 1993 onwards and outpatient visits from 1995 onwards) and from the DPCR (from 1995 onwards) [30]. Additionally, from the DNPR, repeated codes for physician drug prescriptions redeemed at pharmacies and recorded in accordance with the Anatomical Therapeutic Chemical Classification System (ATC) [32] were obtained the one years prior to the index date. To ensure condition chronicity, medical conditions and drug prescriptions were limited to those occurring for the first time at least six months prior to the index date.

Multimorbidity was defined as two or more chronic conditions in an individual according to the definition by the World Health Organization (WHO) [1]. Medical conditions were identified based on the algorithm from the Danish Multimorbidity Index by Prior et al., which operates with 39 medical conditions and/or related redeemed drug prescriptions [33] (Appendix 1). The algorithm was developed for register-based research, based on established multimorbidity indices. It recognizes that medical conditions must be present simultaneously to emerge as multimorbidity and that some chronic conditions may be resolved.

### Existing outpatient trajectories

All outpatient contacts were labelled with a disease category from the Danish Multimorbidity Index, which served as proxies of outpatient trajectories. This involved 31 medical conditions in nine outpatient trajectories (circulatory, endocrinal, pulmonary, gastrointestinal, urogenital, musculoskeletal, hematologic, neurologic system, and cancer) [33] (Appendix 1). An outpatient trajectory was defined as at least two hospital outpatient contacts for the same medical condition within 12 months. Thus, from the index date, we searched back in time for an outpatient appointment in 2017 and an additional encounter within 12 months for the same condition.

### New outpatient trajectories and related contacts

**Fig. 1:**
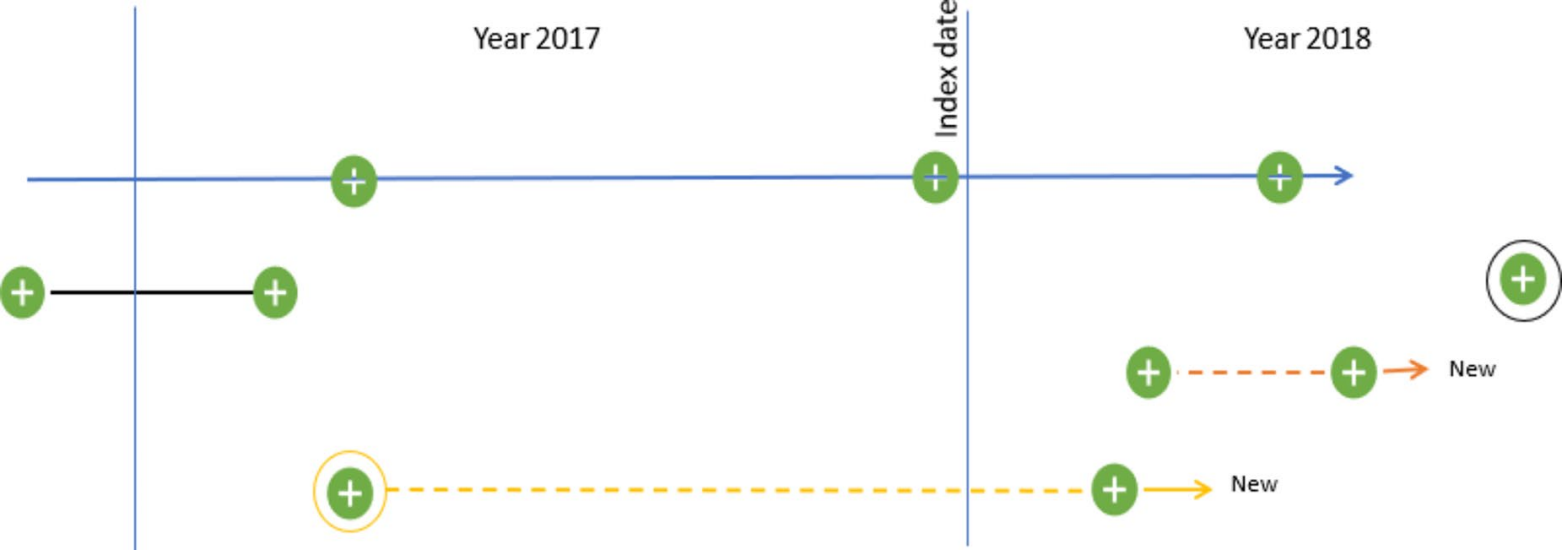
Example of current and new trajectories The colored lines represent trajectories, and the green dots represent contacts to outpatient clinics. In this example, the patient was in two trajectories in 2017 (black and blue solid lines). The orange and yellow solid lines represent new trajectories, as they both involve a second contact for the same chronic condition within a 12-month period. Contacts were estimated for only new trajectories

Being in a new hospital outpatient trajectory was prospectively determined from January 1, 2018, and 12 months onwards. We defined a new hospital outpatient trajectory as two or more outpatient contacts for the same medical condition within a 12-month interval. Thus, we identified the first outpatient contact occurring in 2018 and additional contacts within one year from the date of the first contact; this was done both forward and backwards in time (Fig. 1). After establishing new trajectories, we counted the outpatient contacts in 2018 that were related to these new trajectories.

### Exposure variables

The number of chronic conditions was counted for the 39 medical conditions listed in the Danish Multimorbidity Index, and these counts were grouped into 2, 3, 4, and ≥5 chronic conditions. From Statistics Denmark, we obtained information on gender, age, educational level, country of origin, civil status, population density, labor market attachment, and household income at the index date. Age was divided into <50, 50–59, 60–69, 70–79, and ≥80 years. Information on educational level was categorized in accordance with the International Standard Classification of Education (ISCED) 2011 into groups of <10 years, 10–15 years, and >15 years of education [34]. Ethnicity was categorized into western and non-western countries in accordance with the approach of Statistics Denmark [26]. Population density was based on registered place of residence and divided into <5,000, 5,000–99,999, and >100,000 inhabitants per town. Civil status was dichotomized into living with a partner or living alone. Labor market attachment was categorized into employed, unemployed (incl. retirement), or studying [26]. Household income was estimated for the year 2017 and divided into <20,000, 20,000–34,999, 35,000–49,999, and >50,000 EUR.

Directed acyclic graphs (DAGs) were used to illustrate associations and find relevant variables to adjust for. Information on disease severity was unavailable in the registers, instead we used the number of chronic conditions as a proxy. As educational level is often linked with household income and labor market attachment, educational level was selected as a common measure for socioeconomic status to adjust for. The number of contacts with general practice and health behavior were identified as intermediate factors and colliders and were not adjusted for.

### Statistical analysis

We expected the initiation of new outpatient trajectories to vary by existing use of outpatient trajectories. Therefore, we stratified all analyses by the number of existing trajectories (0, 1, 2, and ≥3).

**Fig. 2:**
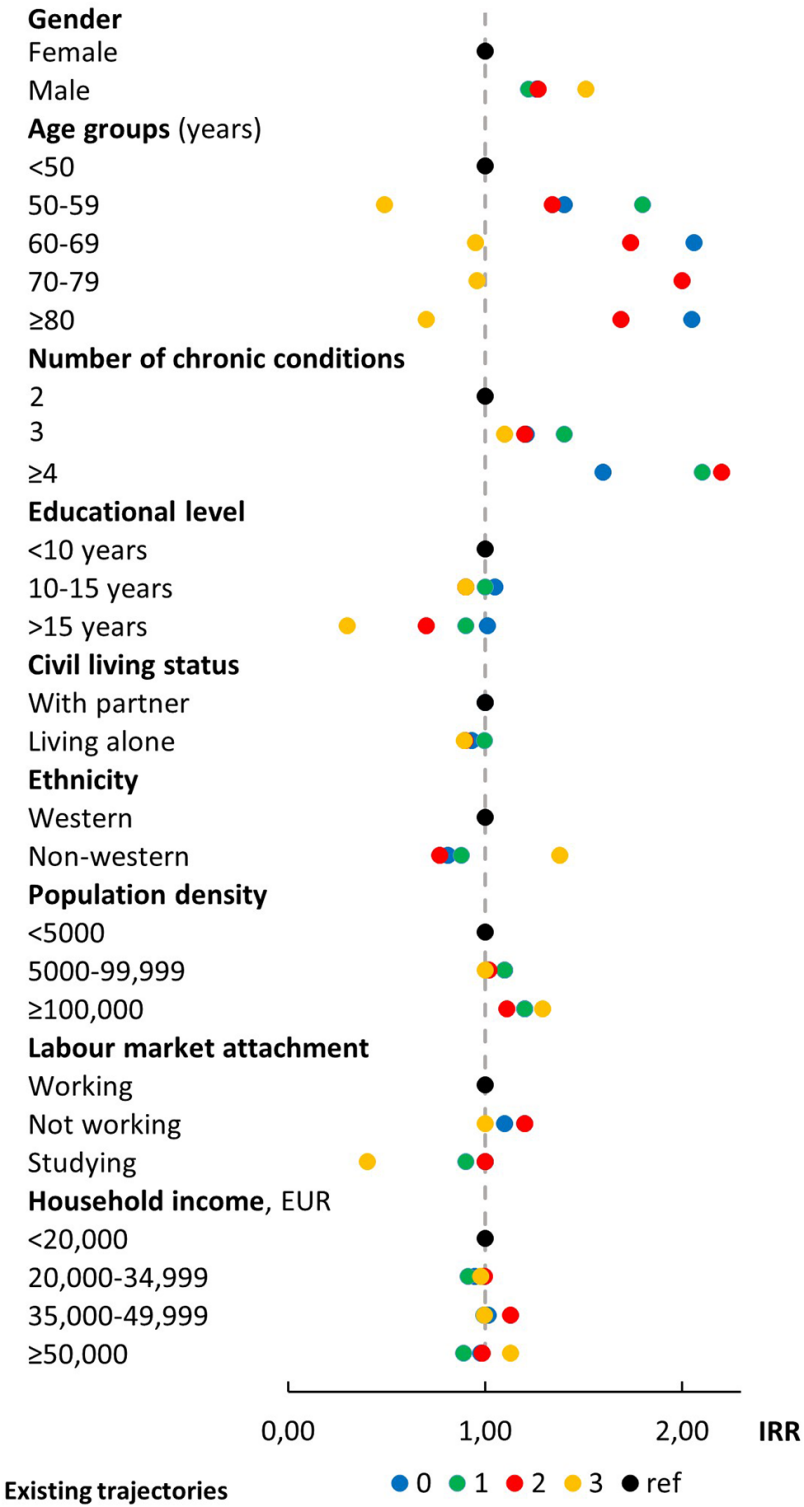
Determinants for having contact in new outpatient trajectories among individuals with multimorbidity according to existing number of trajectories. **IRR**: incidence rate ratio

Descriptive statistics using counts with percentages and medians with interquartile intervals were applied to obtain baseline characteristics of the population. Person-time was calculated, starting from January 1, 2018, until death, emigration, or end of follow-up on December 31, 2018, whichever came first. Person-time for periods of hospitalization were subtracted from the length of observation time for each individual. Incidence rates (IR) and incidence rate ratios (IRR) with 95% confidence intervals (CI) for entering a new outpatient trajectory were estimated in unadjusted and adjusted Poisson regression models for each exposure. Adjusted IRRs for outpatient contacts for those entering a new trajectory were displayed in a forest plot to depict each exposure variable for each of the four existing trajectory categories (0, 1, 2, and ≥3) (Fig. 2) (Appendix 2). Model assumption of linearity was evaluated with a scatterplot for each exposure variable against log-odds of events to check that the rates were constant within covariate patterns. From assessing model assumptions, age showed a parable shaped association and was presented as a grouped exposure to account for the effect that new outpatient utilization may decrease when a person reaches a high age. All data analyses were performed in Stata statistical software, version 17.

## Results

### Participants and descriptive data

We identified 1,321,003 Danish adults with multimorbidity. Tables 1 and 2 show the characteristics of the study population. Chronic conditions related to the circulatory system were most prevalent (77.3-92.6%). Within each disease category, the frequency of chronic conditions rose in concordance with the number of existing trajectories, except for mental health diseases. A little less than half (41.8%) of the individuals with no existing trajectories had two chronic conditions, whereas the majority (82.8%) of individuals in ≥3 trajectories had five or more chronic conditions (Table 1).

**Table 1:** The disease distribution in adults living with multimorbidity in Denmark on 1 January 2018 according to number of existing trajectories

Categories and diagnoses	Existing outpatient trajectories									
	All		0		1		2		≥ 3	
	*N*	*%*	*N*	*%*	*N*	*%*	*N*	*%*	*N*	*%*
**Total**	1,321,003	100	974,147	100	298,981	100	42,856	100	5,019	100
**Circulatory system**	**1,020,746**	**77.3**	**752,481**	**77.3**	**227,796**	**76.2**	**35,819**	**83.6**	**4,650**	**92.6**
Hypertension	796,033	60.3	588,181	60.4	175,843	58.8	28,218	65.8	3,791	75.5
Dyslipidemia	375,603	28.4	287,064	29.5	76,430	25.6	10,874	25.4	1,235	24.6
Ischemic heart disease	226,702	17.2	151,985	15.6	59,737	20.0	12,846	30.0	2,134	42.5
Atrial fibrillation	142,040	10.8	88,710	9.1	42,229	14.1	9,480	22.1	1,621	32.3
Heart failure	67,021	5.1	37,357	3.8	22,067	7.4	6,283	14.7	1,314	26.2
Peripheral artery occlusive disease	86,385	6.5	53,655	5.5	25,330	8.5	6,246	14.6	1,154	23.0
Stroke	131,702	10.0	95,654	9.8	29,691	9.9	5,563	13.0	794	15.8
**Endocrine system**	**384,537**	**29.1**	**255,110**	**26.2**	**104,515**	**35.0**	**21,528**	**50.2**	**3,384**	**67.4**
Diabetes mellitus	237,883	18.0	150,728	15.5	69,393	23.2	15,179	35.4	2,583	51.5
Thyroid disorder	128,949	9.8	88,812	9.1	33,052	11.1	6,202	14.5	883	17.6
Gout	56,543	4.3	36,721	3.8	15,017	5.0	3,967	9.3	838	16.7
**Pulmonic system and allergy**	**293,791**	**22.2**	**214,565**	**22.0**	**65,482**	**21.9**	**11,865**	**27.7**	**1,879**	**37.4**
Chronic pulmonary disease	203,839	15.4	143,827	14.8	48,634	16.3	9,738	22.7	1,640	32.7
Allergy	138,526	10.5	107,314	11.0	26,852	9.0	3,845	9.0	515	10.3
**Urogenital system**	**136,041**	**10.3**	**85,131**	**8.7**	**39,226**	**13.1**	**9,693**	**22.6**	**1,991**	**39.7**
Chronic kidney disease	36,242	2.7	15,447	1.6	13,807	4.6	5,520	12.9	1,468	29.2
Prostate disorder	105,840	8.0	71,774	7.4	27,925	9.3	5,297	12.4	844	16.8
**Gastrointestinal system**	**225,673**	**17.1**	**152,344**	**15.6**	**60,569**	**20.3**	**11,066**	**25.8**	**1,694**	**33.8**
Ulcer/chronic gastritis	77,161	5.8	55,003	5.6	17,968	6.0	3,607	8.4	583	11.6
Chronic liver disease	34,395	2.6	18,219	1.9	13,171	4.4	2,593	6.1	412	8.2
Inflammatory bowel disease	35,942	2.7	19,773	2.0	13,313	4.5	2,492	5.8	364	7.3
Diverticular disease of intestine	100,141	7.6	72,534	7.5	22,933	7.7	4,037	9.4	637	12.7
**Musculoskeletal**	**483,638**	**36.6**	**341,403**	**35.1**	**118,503**	**39.6**	**20,779**	**48.5**	**2,953**	**58.8**
Connective tissue disorders	81,671	6.2	43,096	4.4	30,648	10.3	6,822	15.9	1,105	22.0
Osteoporosis	137,673	10.4	88,895	9.1	39,910	13.3	7,765	18.1	1,103	22.0
Painful condition	356,233	27.0	260,987	26.8	79,730	26.7	13,510	31.5	2,006	40.0
**Hematological**	**94,724**	**7.2**	**59,517**	**6.1**	**28,179**	**9.4**	**5,909**	**13.8**	**1,119**	**22.2**
HIV/AIDS	4,848	3.7	335	< 1	3,957	1.3	488	1.1	68	1.4
Anemias	90,002	6.8	59,186	6.1	24,313	8.1	5,447	12.7	1,056	21.0
**Neurological**	**452,727**	**34.3**	**305,810**	**31.4**	**122,462**	**41.0**	**21,522**	**50.2**	**2,933**	**58.4**
Vision problems	198,345	15.0	134,279	13.8	52,268	17.5	10,208	23.8	1,590	31.7
Hearing problems	201,363	15.2	133,289	13.7	56,540	18.9	10,176	23.7	1,358	27.1
Migraine	41,826	3.2	33,181	3.4	7,807	2.6	780	1.8	58	1.2
Epilepsy	37,803	2.9	24,452	2.5	11,173	3.7	1,917	4.5	261	5.2
Parkinson’s disease	8,698	6.6	4,005	< 1	3,919	1.3	681	1.6	93	1.9
Multiple sclerosis	10,553	8.0	4,399	< 1	5,441	1.8	658	1.5	55	1.1
Neuropathies	32,543	2.6	17,629	18.1	11,937	4.0	2,572	6.0	405	8.0
**Cancers**	**114,704**	**8.7**	**41,508**	**4.2**	**58,443**	**19.5**	**12,931**	**30.2**	**1,822**	**36.3**
**Mental health**	**314,456**	**23.8**	**246,337**	**25.3**	**59,041**	**19.7**	**8,075**	**18.8**	**1,003**	**20.0**
Mood/stress-related/anxiety disorders	50,722	3.8	39,093	4.0	10,150	3.4	1,319	3.1	160	31.9
Psychological distress	188,826	14.3	146,056	15.0	36,800	12.3	5,273	12.3	697	13.9
Alcohol problems	21,016	1.6	16,275	1.7	4,034	1.3	638	1.5	69	1.4
Substance abuse	4,986	< 1	4,369	< 1	551	< 1	61	< 1	5	< 1
Anorexia/bulimia	1,546	< 1	1,336	< 1	184	< 1	23	< 1	< 5	-
Bipolar affective disorder	19,823	1.5	16,359	1.7	3,081	1.0	359	< 1	25	< 1
Schizophrenia/schizoaffective disorder	21,156	1.6	17,927	1.8	2,906	< 1	298	< 1	25	< 1
Dementia	35,512	26.9	29,483	3.0	5,332	1.8	626	1.5	71	1.4
**Number of chronic conditions**										
2	492,276	37.3	406,913	41.8	80,853	27.0	4,402	10.3	108	2.2
3	320,977	24.3	244,727	25.1	69,550	23.3	6,438	15.0	262	5.2
4	207,144	15.7	145,487	14.9	53,776	18.0	7,389	17.2	492	9.8
≥ 5	300,606	22.8	177,020	18.2	94,802	31.7	24,627	57.5	4,157	82.8

**Table 2:** Sociodemographic characteristics in adults living with multimorbidity in Denmark on 1 January 2018 according to number of existing trajectories

Variables	Existing outpatient trajectories									
	**All**		**0**		**1**		**2**		≥ 3	
	*N*	*%*	*N*	*%*	*N*	*%*	*N*	*%*	*N*	*%*
**Total**	1321003	100	974147	100	298981	100	42856	100	5019	100
**Gender**, female	718801	54.4	541011	55.5	155189	51.9	20478	47.8	2123	42.3
**Age groups**, years										
< 50	187,442	14.2	142,302	14.6	41,246	13.8	3,687	8.6	207	4.1
50–59	222,277	16.8	168,137	17.3	47,932	16.0	5,641	13.2	567	11.3
60–69	321,670	24.4	237,383	24.4	72,201	24.2	10,818	25.2	1,268	25.3
70–79	367,817	27.8	260,321	26.7	90,123	30.1	15,271	35.6	2,102	41.9
≥ 80	221,786	16.8	165,996	17.0	47,477	15.9	7,438	17.4	875	17.4
**Educational level**, years										
< 10	462,469	35.0	344,542	35.4	100,981	33.8	15,115	35.3	1,831	36.5
10–15	763,988	57.8	560,392	57.5	175,954	58.9	24,784	57.8	2,858	56.9
≥ 16	66,329	5.0	47,762	4.9	16,254	5.4	2,104	4.9	209	4.2
Missing	28,217	2.1	21,451	2.2	5,792	1.9	853	2.0	121	2.4
**Civil status**, living alone	543945	41.2	407005	41.8	117988	39.5	16912	39.5	2040	40.7
**Ethnicity**										
Western	1,255,886	95.1	926,724	95.1	283,707	94.9	40,672	94.9	4,787	95.3
Non-western	64,526	4.9	46,961	4.8	15,165	5.1	2,168	5.1	232	4.6
Missing	591	0.0	462	0.1	109	0.0	16	0.0	-	-
**Population density**										
< 5000	595,155	45.1	447,190	45.9	128,578	43.0	17,440	40.7	1,947	38.8
5000-99,999	488,814	37.0	362,857	37.3	108,990	36.5	15,215	35.5	1,752	34.9
≥ 100,000	237,019	17.9	164,088	16.8	61,413	20.5	10,201	23.8	1,320	26.3
Missing	15	0.0	12	0.0	< 5	-	< 5	-	< 5	-
**Labor market attachment**										
Working	396,557	30.0	301,233	30.9	85,640	28.6	9,007	21.0	677	13.5
Not working	901,440	68.2	655,492	67.3	208,301	69.7	33,344	77.8	4,306	85.8
Studying	22,872	1.7	17,304	1.8	5,027	1.7	505	1.2	36	0.7
Missing	134	0.0	118	0.0	13	0.0	< 5	-	< 5	-
**Household income**, EUR										
< 20,000	31,241	2.4	23,816	2.4	6,553	2.2	790	1.8	82	1.6
20,000–34,999	331,773	25.1	248,495	25.5	70,912	23.7	10,971	25.6	1,395	27.8
45,000–49,999	322,052	24.4	233,063	23.9	75,398	25.2	12,030	28.1	1,561	31.1
≥ 50,000	635,926	48.1	468,765	48.1	146,118	48.9	19,065	44.5	1,981	39.5
Missing	11	0.0	8	0.0	< 5	-	< 5	-	< 5	-

More than half of the study population with no existing trajectory were women (55.5%), and 42.3% of the cohort in ≥3 existing trajectories were women. Below age 80 years, increasing age was associated with being in more existing trajectories (Table 2). Residents living in high-density areas accounted for 17.9% of the study population. This proportion increased in concordance with increasing number of existing trajectories (16.8-26.6%). The majority (68.2%) had no labor marked attachment; this percentage rose in concordance with increasing number of existing trajectories (67.3-85.8%) (Table 2).

### The rate of initiating new trajectories

Table 3 shows the rate of newly initiated trajectories for one person-year. The highest incidence of entering a new trajectory was seen for individuals with ≥4 chronic conditions and no existing trajectories and for individuals with an annual household income <20,000 euros and ≥3 existing trajectories. The lowest rate of a new trajectory was seen in individuals with two chronic conditions and age <50 years. Overall seen, the lowest IRs (≤0.10) were seen in individuals with two chronic conditions across the stratified groups (Table 3).

**Table 3:** Incidence rate per person-year and relative risk of new outpatient trajectories according to number of existing outpatient trajectories

	Existing outpatient trajectories											
	**0**			**1**				**2**		≥**3**		
	**Risk of entering new trajectories**											
	*Crude*		Adj.*	*Crude*		Adj.*	*Crude*		Adj.*	*Crude*		Adj.*
	*IR*	IRR (95%CI)	IRR (95%CI)	*IR*	IRR (95%CI)	IRR (95%CI)	*IR*	IRR (95%CI)	IRR (95%CI)	*IR*	IRR (95%CI)	*IRR (*95%CI)
**Gender**												
Female Male	0.13 0.16	1 (ref) 1.2 (1.21–1.23)	1 (ref) 1.2 (1.21–1.23)	0.13 0.15	1 (ref) 1.2 (1.17–1.21)	1 (ref) 1.2 (1.17–1.21)	0.14 0.16	1 (ref) 1.2 (1.15–1.27)	1 (ref) 1.2 (1.15–1.27)	0.16 0.19	1 (ref) 1.2 (1.01–1.34)	1 (ref) 1.2 (1.01–1.34)
**Age groups**, years												
<50 50–59 60–69 70–79 ≥80	0.09 0.11 0.14 0.18 0.16	1 (ref) 1.2 (1.18–1.23) 1.6 (1.56–1.63) 2.1 (2.01–2.09) 1.9 (1.85–1.93)	1 (ref) 1.2 (1.16–1.22) 1.6 (1.54–1.60) 2.0 (1.98–2.06) 1.9 (1.86–1.94)	0.06 0.10 0.14 0.18 0.17	1 (ref) 1.6 (1.53–1.69) 2.3 (2.15–2.35) 2.9 (2.73–2.97) 2.7 (2.57–2.81)	1 (ref) 1.6 (1.52–1.67) 2.2 (2.13–2.33) 2.8 (2.71–2.95) 2.7 (2.57–2.81)	0.07 0.11 0.15 0.18 0.17	1 (ref) 1.6 (1.35–1.81) 2.1 (1.84–2.40) 2.5 (2.22–2.87) 2.5 (2.14–2.80)	1 (ref) 1.5 (1.33–1.79) 2.1 (1.80–2.35) 2.5 (1.63–2.81) 2.4 (2.10–2.76)	0.14 0.12 0.18 0.19 0.17	1 (ref) 0.8 (0.53-1.29) 1.3 (0.87-1.92) 1.4 (0.93-2.01) 1.2 (0.81-1.84)	1 (ref) 0.8 (0.54-1.33) 1.3 (0.88-1.97) 1.4 (0.94-2.06) 1.3 (0.82-1.89)
**Number of chronic conditions**												
2 3 ≥4	0.10 0.13 0.20	1 (ref) 1.4 (1.35–1.39) 2.0 (2.01–2.06)	1 (ref) 1.3 (1.27–1.31) 1.9 (1.82–1.87)	0.07 0.11 0.19	1 (ref) 1.5 (1.46–1.57) 2.8 (2.68–2.84)	1 (ref) 1.4 (1.34–1.44) 2.3 (2.27–2.41)	0.06 0.09 0.18	1 (ref) 1.4 (1.21–1.63) 2.9 (2.59–3.32)	1 (ref) 1.3 (1.13–1.52) 2.5 (2.20–2.84)	0.05 0.09 0.18	1 (ref) 2.0 (0.74-5.13) 3.8 (1.57–9.12)	1 (ref) 1.8 (0.70-4.83) 3.3 (1.37-8.00)
**Educational level**, years												
<10 10–15 >15	0.14 0.14 0.14	1 (ref) 9.7 (0.96-0.98) 0.95 (0.93-0.98)	1 (ref) 1.0 (1.03–1.06) 1.1 (1.04–1.10)	0.15 0.13 0.11	1 (ref) 0.9 (0.89-0.93) 0.8 (0.71-0.79)	1 (ref) 1.0 (0.97-1.02) 0.9 (0.86-0.95)	0.17 0.14 0.13	1 (ref) 0.9 (0.81-0.90) 0.8 (0.66-0.85)	1 (ref) 0.9 (0.85-0.95) 0.8 (0.73-0.94)	0.19 0.18 0.07	1 (ref) 0.9 (0.79-1.05) 0.4 (0.22-0.65)	1 (ref) 0.9 (0.79-1.06) 0.4 (0.23-0.66)
**Civil living status**												
With partner Living alone	0.14 0.14	1 (ref) 0.97 (0.96-0.98)	1 (ref) 0.9 (0.93-0.95)	0.14 0.14	(ref) 1.0 (1.01–1.05)	1 (ref) 1.0 (0.99-1.03)	0.15 0.15	1 (ref) 1.0 (0.92-1.02)	1 (ref) 1.0 (0.91-1.01)	0.18 0.17	(ref) 0.9 (0.80-1.06)	1 (ref) 0.9 (0.81-1.08)
**Ethnicity**												
Western Non-western	0.14 0.12	1 (ref) 0.8 (0.81-0.85)	1 (ref) 0.8 (0.81-0.85)	0.14 0.11	1 (ref) 0.8 (0.78-0.86)	1 (ref) 0.8 (0.78-0.86)	0.15 0.13	1 (ref) 0.8 (0.73-0.94)	1 (ref) 0.8 (0.73-0.94)	0.17 0.17	(ref) 1.0 (0.69-1.34)	(ref) 1.0 (0.69-1.34)
**Population density**												
<5000 5000-99,999 ≥100,000	0.14 0.14 0.15	1 (ref) 1.0 (1.02–1.05) 1.1 (1.06–1.09)	1 (ref) 1.0 (1.03–1.06) 1.1 (1.09–1.12)	0.14 0.14 0.14	1 (ref) 1.0 (1.02–1.07) 1.0 (1.00-1.04)	1 (ref) 1.0 (1.01–1.06) 1.1 (1.06–1.12)	0.15 0.15 0.15	1 (ref) 1.0 (0.93-1.05) 1.0 (0.94-1.05)	1 (ref) 1.0 (0.95-1.06) 1.0 (0.96 − 1.10)	0.18 0.17 0.17	1 (ref) 1.0 (0.82 − 1.3) 0.9 (0.78-1.11)	1 (ref) 1.0 (0.84-1.17) 1.0 (0.83-1.19)
**Labor market attachment**												
Working Not working Studying	0.11 0.16 0.09	1 (ref) 1.5 (1.43–1.47) 0.8 (0.78–0.87)	1 (ref) 1.1 (1.07–1.11) 0.9 (0.87-0.97)	0.09 0.16 0.08	1 (ref) 1.7 (1.70–1.78) 0.9 (0.84-1.03)	1 (ref) 1.2 (1.15–1.22) 1.1 (0.98-1.21)	0.10 0.17 0.09	1 (ref) 1.6 (1.47–1.70) 0.9 (0.68-1.22)	1 (ref) 1.2 (1.07–1.25) 0.9 (0.68-1.31)	0.14 0.18 0.09	1 (ref) 1.3 (1.06–1.65) 0.6 (0.20-1.96)	(ref) 1.1 (0.88–1.40) 0.7 (0.22-2.19)
**Household income**, EUR												
<20,000 20,000–34,999 35,000–49,999 ≥50,000	0.11 0.15 0.16 0.13	1 (ref) 1.3 (1.27–1.38) 1.5 (1.38–1.49) 1.2 (1.13–1.22)	1 (ref) 1.0 (0.95-1.04) 1.1 (1.03–1.12) 1.1 (1.01–1.10)	0.11 0.15 0.16 0.12	1 (ref) 1.4 (1.27–1.48) 1.5 (1.34–1.55) 1.1 (1.00-1.16)	1 (ref) 0.9 (0.87-1.02) 1.0 (0.91-1.07) 0.9 (0.86 − 1.00)	0.12 0.16 0.17 0.13	(ref) 1.3 (1.05–1.59) 1.4 (1.14–1.73) 1.1 (0.88-1.34)	1 (ref) 1.1 (0.85-1.34) 1.1 (0.91-1.43) 1.1 (0.84-1.32)	0.22 0.17 0.18 0.17	1 (ref) 0.8 (0.47–1.33) 0.9 (0.51–1.43) 0.8 (0.45–1.28)	1 (ref) 0.7 (0.38–1.14) 0.7 (0.39–1.18) 0.7 (0.41–1.19)

IR: Incidence rates. IRR: Incidence rate ratios. All figures are rounded. *Gender and ethnicity were not adjusted for covariates. Age was adjusted for gender and ethnicity. Number of chronic conditions was adjusted for age, gender, educational level, and ethnicity. Educational level was adjusted for age, gender, number of chronic conditions, and ethnicity. Civil status was adjusted for age. Population density was adjusted for age, educational level, and ethnicity. Labor market attachment was adjusted for age, gender, number of chronic conditions, educational level, and ethnicity. Household income was adjusted for age, gender, number of chronic conditions, educational level, and ethnicity

Adjusted relative incidence rates (IRR) showed that male gender and increasing number of chronic conditions were determinants for entering a new trajectory across all groups with existing trajectories. In addition, the IRR of having high numbers of existing trajectories increased in concordance with the number of chronic conditions. For example, those with ≥4 chronic conditions and ≥3 existing trajectories had a 3.3 (95%CI: 1.37-8.00) times higher rate of entering a new trajectory compared to 1.9 (95%CI: 1.82–1.87) times higher in those with ≥4 chronic conditions and no existing trajectory.

A high educational level was a determinant for those with no existing trajectories. Conversely, a lower rate was seen in those with 1, 2, or ≥3 existing trajectories and a high educational level.

Individuals with ≥3 existing trajectories and a high educational level had a 0.40 (95%CI: 0.23–0.66) times higher rate of a new trajectory compared to peers with a low educational level.

For those in 0, 1, or 2 existing outpatient trajectories, rising age, western ethnicity, and no labor market attachment increased the rate of entering a new trajectory. Notably, age showed a parabolic-shaped association, which broke at age ≥80 years. A population density ≥5,000 residents per town increased the rate of a new trajectory among people in no and 1 existing outpatient trajectory. Living with a partner and having high household income increased the rate of a new trajectory in those with no existing trajectories (Table 3).

### The rate of contacts in new trajectories

Figure 2 displays the rate of outpatient contacts related to initiation of new trajectories. Age and number of chronic conditions showed the highest increased risks of new contacts across the stratified groups. Age group showed parabolic associations; higher IRRs were seen for those with 0, 1, or 2 existing trajectories and (slightly lower) for those with ≥3 existing trajectories when we compared the high age group to the reference group below age 50 years.

## DISCUSSION

### Key results

The rates for initiating a new trajectory were highest among individuals with three or more chronic conditions and no existing outpatient trajectories, and among individuals with an annual household income below 20,000 EUR and three or more existing trajectories. A growing number of chronic conditions and male gender increased the rate of having outpatient trajectories initiated. Associations with other variables were modified by the number of existing trajectories for an individual. A low educational level was a determinant at one or more existing trajectories. For those with less than three existing trajectories, high age, western ethnicity, and no work force attachment was associated with higher rates of having a new trajectory initiated. Likewise, increasing age and increasing number of chronic conditions increased the rate of outpatient contact in new trajectories.

### Existing research

Variations in healthcare utilization and associations with the characteristics of individuals with multimorbidity has previously been studied, but little is known about the use of specialized outpatient care in the hospital setting [7, 8, 14, 15, 19, 20, 35], and especially on the determinants for having outpatient trajectories initiated.

Notably, our results showed that men initiated new trajectories at a higher rate than women. In Denmark, GPs are gatekeepers to specialist outpatient care [28]. Female gender is associated with multimorbidity and the care provision in general practice is known to differ by gender as women contact GPs more frequently, although, attendance for a yearly chronic care consultation is the same across gender [11, 36]. Therefore, men are unlikely to be referred to specialist care more often than women. Unless, their disease progression is more advanced, when seeking medical care. Moreover, regular attendance and focus on disease management in general practice (by women) may prevent initiation of specialist care [37, 38]. Applying at least two contacts within a year’s distance as definition for being in an outpatient trajectory resulted in finding those with continual healthcare utilization. In Denmark, women below 65 years of age account for higher use of outpatient clinics more than men, while men from the age of 65 to 80 years constitute a higher provision than women [26].

We found the rate of new trajectories to increase by age, although in a parabolically shaped association as it was less profound for individuals aged ≥80 years. It is well known that ageing is associated with frailty and functional impairment [39, 40] and acceleration of primary healthcare use [36], while the utilization of specialist care decreases [9]. This could be due to several factors. First, many hospital visits may seem unwarranted at a high age. Second, the healthy elderly population may receive fewer new trajectories and contacts. Third, initiation of new outpatient services might be prioritized less for the elderly, or their needs for specialist care might be met in the context of geriatric care.

Adding chronic conditions increased the rate of new trajectories. This is consistent with previous studies, which have shown an association between degree of multimorbidity and specialized healthcare utilization [9, 14, 15]. Chronic conditions tend to cluster together, and disease patterns differ in complexity and healthcare utilization [41]; this will influence the initiation of outpatient care. However, wide and unwarranted variations in healthcare provision have been reported in the Wennberg Atlas Project, where variations could not be explained by type or severity of illness or by patient preferences [42]. Despite reports of care fragmentation for specialist outpatients [43], rare attempts have been made to align trajectories and foster collaboration across specialties to reduce the number of outpatient trajectories [3, 35]. Also, it remains unknown how an individual with multimorbidity is best included in several trajectories of specialist healthcare.

Our results showed higher rates of entering a new trajectory for those with low education or unemployment. Previous studies have established an inverse socioeconomic gradient in healthcare utilization [15, 19, 20] and a recent review concluded that the most disadvantaged individuals often have lower probability of visiting specialists [20]. Unemployment may not be the only cause of socioeconomic inequality in our study; it may simply reflect that our study participants had reached pension age. However, this cannot explain why low education appeared to be a predisposing factor.

### Strengths and limitations

A major strength of this study is the nationwide prospective design based on a comprehensive cohort of Danish adults with multimorbidity. An additional strength was the use of large and complete electronic registers, which reduced the risk of information bias and selection bias. Data from Danish national registers contain complete information on clinical services provided and are collected consecutively with the purpose of clinical surveillance and remuneration, and these data are known to have high validity and little loss of follow-up [28, 29]. Healthcare utilization is not affected by health insurance coverage as the Danish healthcare system offers universal access to healthcare and public hospitals. This makes the Danish health registers highly appropriate for research purposes.

A trajectory was restricted to a minimum of two outpatient visits for the same condition within 12 months to ensure consistency in the definition of being in a trajectory. In the analyses, we deducted hospitalization time and accounted for loss to follow-up by death or emigration from person-time followed, to avoid selection bias by attrition. Our analyses were stratified to account for already existing use of outpatient services as this was hypothesized to influence initiation of a new trajectory.

Some limitations should be addressed. This was an observational design, which cannot be used to determine causality. The results were confined to our definitions of both multimorbidity and trajectories with selected chronic conditions. This selection seems in line with the most frequently included chronic conditions in earlier studies and categorisation into disease system groups [44] Since outpatient visits were included for selected health conditions, our results are likely to have underestimated the true number of trajectories and contacts in outpatient clinics. Some could have been lost due to the lack of diagnosis (e.g., in general practice). However, our cohort was identified through hospital attendance during two decades and through medical prescriptions redeemed at pharmacies and recorded in the Danish Multimorbidity Index [33]. Thus, both diagnoses made in the hospital setting and diagnoses based on disease-specific prescriptions in general practice were included. Still, some diagnoses may not have been identified if no prescriptions were given for the medical condition because no register holds records of diagnoses made in general practice.

By looking back in time to establish the state of being in a trajectory, we minimized left-truncated selection bias, which could otherwise have overestimated the incidence and underestimated the prevalence of being in an existing trajectory. Moreover, we restricted to a minimum of two consecutive outpatient visits for the same condition to ensure consistency in the definition of being in a trajectory.

If individuals with short-term follow-up due to hospitalization were systematically different from those with long-term follow-up and had equally many new trajectories initiated after discharge, the resulting incidence density would depend on the combination of individuals and follow-up time. Even so, if we had not reduced person-time in the analyses, hospitalized individuals would have contributed with person-time without being at risk. Residual confounding by unmeasured factors or by the grouping of variables cannot be ruled out. Register-based research is limited to the data and level of detail recorded, and no data were available on condition severity, health behavior, lifestyle factors, or being in a trajectory. However, we conditioned our analysis on the number of chronic conditions as a proxy for health status. Since health behavior was assessed as an intermediate factor and collider in our DAGs, health behavior needed no conditioning in the analysis. As we deducted periods of hospitalization from person-time, some individuals were followed for a shorter time.

### Clinical implications

Individuals with multimorbidity often encounter many different healthcare professionals across multiple provider settings [35, 45]. Knowledge about characteristics associated with initiation of new trajectories may reveal disparities in the provision of outpatient care and enable clinicians to identify individuals eligible for intensified utilization of healthcare services, who may require special attention. Such information will enable clinicians to offer better planned outpatient services and more coherent care. Better utilization will allow for more accurate allocation of resources and alignment of several different care trajectories. The reconfiguration of healthcare supporting the management of multimorbidity is urgently needed, due to increasing multimorbidity. Our results may guide reconfiguration by directing efforts towards those who often initiate new outpatient trajectories. As this study was population-based and studied the initiation of new outpatient trajectories among adults with multimorbidity, which can be observed in other health care systems, the findings are generalizable to other healthcare settings that resemble the Danish system i.e. publicly funded healthcare systems situated in Scandinavia and Northern Europe.

## Conclusion

This novel research investigated determinants for initiating new outpatient trajectories for individuals with multimorbidity. One of the most noticeable results was that the rate of new trajectories was higher for men than for women. Moreover, we found that the rate of new trajectories increased with age, increasing number of chronic conditions, and low socioeconomic status (i.e., those with the highest needs for care). These rates were modified by the number of already existing outpatient trajectories. High age and high number of chronic conditions increased the rate of having outpatient contacts.

## Electronic supplementary material

Below is the link to the electronic supplementary material.

Supplementary Material 1

## Data Availability

The data that support the findings of this study were used under license from Statistical Denmark and are not publicly available. Similar data are available for researchers upon application to Statistics Denmark (www.dst.dk/en).

## Abbreviations

ATC: Anatomical Therapeutic Chemical Classification System
CI: Confidence interval
CRS: Danish Civil Registration System
DAGs: Directed acyclic graphs
DNPR: the Danish National Prescription Registry
DPCR: the Danish Psychiatric Central Register
GP: General practitioner
IQI: Interquartile interval
IR: Incidence rate
IRR: Incidence rate ratio
NPR: Danish National Patient Register
SES: Socioeconomic status
