# Supplementary material for: Multimorbidity and determinants for initiating outpatient trajectories: A population-based study
Source: BMC Public Health. 2023 Apr 21;23:739. doi: 10.1186/s12889-023-15453-w (PMC10120141; doi:10.1186/s12889-023-15453-w)
Supplement: Supplementary file 1 — Supplementary Material 1 [file 12889_2023_15453_MOESM1_ESM.docx]

| **Appendix 1.** Algorithm for assessing physical and mental multimorbidity | | | | | | | |
| --- | --- | --- | --- | --- | --- | --- | --- |
| **Trajectory category** | **Disease group** | **Origin^a^** | **Coding definition^b^** | **Diagnosis codes (ICD-10)** | **Diagnosis time frame** | **Drug codes (ATC)** | **Prescription time frame** |
| **Circulatory system** | Hypertension | a, b | Diagnosis AND/OR prescriptions of antihypertensives if not ischemic heart disease or heart failure (or kidney disease: only diuretics) | I10-I13, I15 | Ever | C02, C04, C07, C08, C09, C03 | Twice last year |
|  | Dyslipidemia | b | Diagnosis AND/OR drug prescription for lipid-lowering drugs if not ischemic heart disease | E78 | Last two years | C10 | Twice last year |
|  | Ischemic heart disease | a, b | Diagnosis AND/OR prescription for antianginal drug | I20-I25 | Ever | C01DA | Twice last year |
|  | Atrial fibrillation | a, b | Diagnosis | I48 | Ever |  |  |
|  | Heart failure | a, b | Diagnosis | I50 | Ever |  |  |
|  | Peripheral artery occlusive disease | a, b | Diagnosis | I70-I74 | Ever |  |  |
|  | Stroke | a, b | Diagnosis | I60-I64, I69 | Ever |  |  |
| **Endocrine system** | Diabetes mellitus | a, b | Diagnosis AND/OR prescription of antidiabetics | E10-E14 | Ever | A10A, A10B | Twice last year |
|  | Thyroid disorder | a, b | Diagnosis AND/OR prescription of thyroid therapy drugs | E00-E05, E061-E069, E07 | Last two years | H03 | Twice last year |
|  | Gout | b | Diagnosis | E79, M10 | Ever |  |  |
| **Pulmonary system and allergy** | Chronic pulmonary disease | a, b | Diagnosis AND/OR prescription for obstructive airway disease drugs | J40-J47, J96 | Ever | R03 | Twice last year |
|  | Allergy | b | Diagnosis AND/OR prescription for non-sedative antihistamines AND/OR nasal antiallergics |  | Ever | R06AX, R06AE07, R06AE09, R01AC, R01AD | Twice last year |
| **Gastrointestinal system** | Ulcer/chronic gastritis | a, b | Diagnosis | K221, K25-K28, K293-K295 | Ever |  |  |
|  | Chronic liver disease | a, b | Diagnosis | B16-B19, K70-K74, K766, I85 | Ever |  |  |
|  | Inflammatory bowel disease | a, b | Diagnosis | K50-K51 | Ever |  |  |
|  | Diverticular disease of intestine | a, b | Diagnosis | K57 | Ever |  |  |
| **Urogenital system** | Chronic kidney disease | a, b | Diagnosis | N03, N11, N18-N19 | Ever |  |  |
|  | Prostate disorders | a, b | Diagnosis AND/OR prescription of prostate hyperplasia therapy drugs | N40 | Ever | C02CA, G04C | Twice last year |
| **Musculoskeletal system** | Connective tissue disorders | a, b | Diagnosis | M05-M06, M08-M09, M30-M36, D86 | Ever |  |  |
|  | Osteoporosis | b | Diagnosis AND/OR prescription for osteoporosis drugs | M80-M82 | Ever | M05B, G03XC01, H05AA | Twice last year |
|  | Painful condition | a, b | Repeated prescriptions of analgesics |  |  | N02A,  N02BA51, N02BE, M01A, M02A | Four times last year |
| **Hematological system** | HIV/AIDS | b | Diagnosis | B20-B24 | Ever |  |  |
|  | Anemias | b | Diagnosis | D50-D53, D55-D59, D60-D61, D63-D64 | Last two years |  |  |
| **Cancers** | Cancer | a, b | Diagnosis | C00-C43, C45- C97 | Last five years |  |  |
| **Neurological system** | Vision problem | a, b | Diagnosis | H40, H25, H54 | Ever |  |  |
|  | Hearing problem | a, b | Diagnosis | H90-H91, H931 | Ever |  |  |
|  | Migraine | a, b | Diagnosis AND/OR prescription of specific anti-migraine drugs | G43 | Last two years | N02C | Twice last year |
|  | Epilepsy | a, b | Diagnosis AND prescription of anti-epileptic drugs | G40-G47 | Ever | N03 | Twice last year |
|  | Parkinson's disease |  | Diagnosis | G20-G22 | Ever |  |  |
|  | Multiple sclerosis | a | Diagnosis | G35 | Ever |  |  |
|  | Neuropathies | b | Diagnosis | G50-G64 | Last two years |  |  |
| **Mental health conditions** | Mood, stress-related, or anxiety disorders | a, b | Diagnosis | F32-F34, F40-F48 | Last two years |  |  |
|  | Psychological distress | a, b | Prescription of antidepressants if no other mental disorder |  |  | N06A | Twice last year |
|  | Alcohol problems | a, b | Diagnosis | F101-F109 | Last two years |  |  |
|  | Substance abuse | a, b | Diagnosis | F11-F16, F18-F19 | Last two years |  |  |
|  | Anorexia/bulimia | a | Diagnosis | F50 | Last two years |  |  |
|  | Bipolar affective disorder | a, b | Diagnosis AND/OR prescription of lithium salts | F30-F31 | Ever | N05AN | Twice last year |
|  | Schizophrenia or schizoaffective disorder | a, b | Diagnosis | F20, F25 | Ever |  |  |
|  | Dementia | a, b | Diagnosis AND/OR prescription of anti-dementia drugs | F00-F03, F051, G30 | Ever | N06D | Twice last year |
| **Abbreviations**: ICD-10, International Classification of Diseases, 10^th^ revision; ATC, Anatomical Therapeutic Chemical classification system; HIV, human immunodeficiency virus; AIDS, acquired immunodeficiency syndrome.  **^a^** ICD-10 diagnosis code recorded or redeemed prescription of ATC-coded drug registered within defined time frames (time range: 1995-2019) in national health registers (the Danish National Patient Register, the Danish Diabetes Register, the Danish Cancer Register, the Danish Psychiatric Central Register, and the Danish National Prescription Registry). **^b^** a: Ref: Barnett et al.,^1^ b: Other index (Van den Bussche H. et al., Huber CA et al., Charlson ME et al., and/or Elixhauser A. et al.)  References  1) Barnett K, Mercer SW, Norbury M, Watt G, Wyke S, Guthrie B. Epidemiology of Multimorbidity and implications for health care, research, and medical education: A cross-sectional study. Lancet. 2012;380(9836):37-43.  2) Van den Bussche H, Schon G, Kolonko T, et al. Patterns of ambulatory medical care utilization in elderly patients with special reference to chronic diseases and multimorbidity—results from a claims data based observational study in Germany.  3) Huber CA, Szucs TD, Rapold R, Reich O. Identifying patients with chronic conditions using pharmacy data in Switzerland: An updated mapping approach to the classification of medicines. BMC Public Health. 2013;13:1030-2458-13-1030.  4) Charlson ME, Pompei P, Ales KL, Mackenzie CR. A new method of classifying prognostic comorbidity in longitudinal studies: Development and validation. J Chronic Dis. 1987;40(5):373-383.  5) Elixhauser A, Steiner C, Harris DR, Coffey RM. Comorbidity measures for use with administrative data. Med Care. 1998;36(1):8-27). | | | | | | | |

**Appendix 2.** Adjusted relative risk of contacts in new outpatient trajectories per person-year according to number of existing trajectories

|  | **Number of existing trajectories** | | | | | | | |
| --- | --- | --- | --- | --- | --- | --- | --- | --- |
|  | **0** | | **1** | | **2** | | **3** | |
|  | **Risk of new outpatient contact*** | | | | | | | |
|  | *Adj. IRR* | *95%CI* | *Adj. IRR* | *95%CI* | *Adj. IRR* | *95%CI* | *Adj. IRR* | *95%CI* |
| **Gender** |  |  |  |  |  |  |  |  |
| Female  Male | 1  1.23 | (ref)  (1.225-1.239) | 1  1.18 | (ref)  (1.165-1.190) | 1  1.22 | (ref)  (1.189-1.254) | 1  1.47 | (ref)  (1.362-1.590) |
| **Age groups**, years |  |  |  |  |  |  |  |  |
| <50  50-59  60-69  70-79  ≥80 | 1  1.40  2.06  2.60  2.05 | (ref)  (1.385-1.422)  (2.037-2.085)  (2.571-2.629)  (2.230-2.074) | 1  1.60  2.07  2.40  1.78 | (ref)  (1.555-1.639)  (2.024-2.125)  (2.339-2.453)  (1.739-1.831) | 1  1.39  1.69  1.84  1.47 | (ref)  (1.287-1.496)  (1.580-1.812)  (1.716-1.963)  (1.371-1.584) | 1  .47  .84  .83  .56 | (ref)  (.380-.579)  (.703-.995)  (.701-.982)  (.483-.705) |
| **Number of chronic conditions** | | | | | | | | |
| 2  3  ≥4 | 1  1.21  1.60 | (ref)  (1.205-1.224)  (1.589-1.611) | 1  1.37  2.09 | (ref)  (1.342-1.391)  (2.056-2.120) | 1  1.24  2.22 | (ref)  (1.154-1.338)  (2.089-2.368) | 1  1.10  2.33 | (ref)  (.708-1.707)  (1.595-3.412) |
| **Educational level**, years | | | | | | | | |
| <10  10-15  >15 | 1  1.06  1.01 | (ref)  (1.048-1.061)  (.995-1.024) | 1  .97  .87 | (ref)  (.962-.984)  (.845-.891) | 1  .90  .68 | (ref)  (.872-.921)  (.630-.729) | 1  .94  .29 | (ref)  (.869-1.010)  (.211-.397) |
| **Civil living status** | | | | | | | | |
| With partner  Living alone | 1  .95 | (ref)  (.939-.951) | 1  .98 | (ref)  (.971-.992) | 1  .88 | (ref)  (.856.906) | 1  .94 | (ref)  (.867-1.013) |
| **Ethnicity** | | | | | | | | |
| Western  Non-western | 1  .97 | (ref)  (.951-.981) | 1  1.09 | (ref)  (1.059-1.116) | 1  .86 | (ref)  (.798-.920) | 1  1.63 | (ref)  (1.405-1.896) |
| **Population density** | | | | | | | | |
| <5000  5000-99.999  ≥100.000 | 1  1.06  1.20 | (ref)  (1.056-1.069)  (1.189-1.208) | 1  1.07  1.19 | (ref)  (1.057-1.082)  (1.170-1.203) | 1  1.02  1.12 | (ref)  (.988-1.050)  (1.078-1.153) | 1  1.02  1.37 | (ref)  (.936-1.116)  (1.257-1.503) |
| **Labor market attachment** | | | | | | | | |
| Working  Not working  Under education | 1  1.12  .95 | (ref)  (1.109-1.127)  (.926-.981) | 1  1.22  .91 | (ref)  (1.203-1.240)  (.860-.967) | 1  1.16  .99 | (ref)  (1.109-1.202)  (.846-1.164) | 1  .97  .40 | (ref)  (.868-1.088)  (.196-.798) |
| **Household income**, EUR | | | | | | | | |
| <20,000  20,000-34,999  35,000-49,999  ≥50,000 | 1  .95  1.02  .98 | (ref)  (.928-.970)  (.996-1.041)  (.958-1.00) | 1  .91  .98  .88 | (ref)  (.868-.943)  (.944-1.025)  (.844-.916) | 1  1.02  1.16  1.01 | (ref)  (.910-1.143)  (1.033-1.296)  (.903-1.131) | 1  .93  .94  1.04 | 1 (ref)  (.669-1.298)  (.676-1.307)  (.748-1.441) |

IRR: Incidence rate ratios. All figures are rounded. Gender and ethnicity were not adjusted for covariates. Age was adjusted for gender and ethnicity. Number of chronic conditions was adjusted for age, gender, educational level, and ethnicity. Educational level was adjusted for age, gender, number of chronic conditions, and ethnicity. Civil status was adjusted for age. Population density was adjusted for age, educational level, and ethnicity. Lor market attachment was adjusted for age, gender, number of chronic conditions, educational level, and ethnicity. Household income was adjusted for age, gender, number of chronic conditions, educational level, and ethnicity.
